# Surgical Marking Pen Contamination: Writing a Postoperative Infection Into Your Preoperative Plan

**DOI:** 10.7759/cureus.40007

**Published:** 2023-06-05

**Authors:** Mallorie L Huff, Aaron M Jacobs, Evanie Huang, Marshall G Miles

**Affiliations:** 1 Division of Plastic and Reconstructive Surgery, Department of Surgery, Lehigh Valley Health Network, Allentown, USA; 2 Division of Plastic and Reconstructive Surgery, Department of Surgery, University of South Florida Health Morsani College of Medicine, Tampa, USA

**Keywords:** surgical safety, marking pen, infection prevention and control, surgical site infection (ssi), preoperative marking

## Abstract

Introduction

Preoperative marking is an essential safety practice to prevent "never" events, including wrong site surgery. Moreover, the Joint Commission regulations of the Universal Protocol require that patients be marked to indicate the operative site. Marking typically occurs with a pen or marker, which may be disposable or reusable. Previous studies have demonstrated that methicillin-resistant *Staphylococcus aureus* (MRSA) can survive in the dark, moist, capped environment of the marking pen and thus could plausibly be a nidus for transmission from patient to patient. The Joint Commission has established no increased risk of postoperative infection with these markings. With this study, we aimed to determine the colonization of surgical marking pens in the plastic surgery population.

Methods

Two marking pens from five different attending plastic surgeons at a single institution were cultured in standard fashion for aerobic and anaerobic growth. All pens were used repeatedly in office settings for performing patient markings. Those same ten marking pens were then used to mark incision sites on mock patients. Standard povidone-iodine prepping was then performed in a paint-only fashion over the skin markings, and cultures were again taken. A control group consisted of cultures from five sterile pens from the operating room. Each sterile pen was opened, uncapped, and then swabbed. All twenty-five cultures were analyzed in the hospital laboratory in a blinded fashion.

Results

The five control pens revealed no bacterial growth. Of the 10 direct pen cultures, two samples grew coagulase-negative staphylococci and one culture contained *Pseudomonas aeruginosa*. The 10-patient marked and prepped specimens showed eight negative cultures and two with coagulase-negative staphylococci. Although *Pseudomonas* was detected on standard pen culture, no pseudomonal growth was present in any of the samples after patient marking and prepping with povidone-iodine.

Conclusions

Our findings reaffirm that marking pens may be vehicles for bacterial transmission and expand upon previous studies by describing the presence of bacterial colonization on marking pens even after surgical site preparation with povidone-iodine.

## Introduction

Surgical site infections (SSIs) are a significant postsurgical complication that causes upwards of 290,000 healthcare-associated infections (HAIs) and 8,000 deaths in the United States annually [1]. While considerable efforts have been made to characterize the best practices of SSI prophylaxis and management, there remains significant variation in the practices reported in the literature, with calls for increased study of evidence-based best practices [1].

Preoperative marking is an essential safety practice to prevent "never" events, including wrong site surgery. Broadly, never events, as defined by the National Quality Forum (NQF), comprise 29 events that are divided into seven categories: surgical or invasive procedural events, product or device events, patient protection events, care management events, environmental events, radiologic events, and potential criminal events. Of these categories, wrong site surgery falls under surgical or invasive procedural events [2]. Moreover, the Joint Commission regulations of the Universal Protocol require that patients be marked to indicate the operative site [3]. Marking typically occurs with a pen or marker, which may be disposable or reusable. Previous studies have demonstrated that methicillin-resistant *Staphylococcus aureus* (MRSA) can survive in the dark, moist, capped environment of the marking pen and thus could plausibly be a nidus for transmission from patient to patient [4]. The Joint Commission has described no increased risk of postoperative infection with these markings [3]. Historically, our institution has used a “paint only” approach to the surgical prep after marking to avoid erasing the surgical markings. With this study, we aimed to determine the colonization of surgical marking pens in the plastic surgery population. We hypothesized that reusing marking pens, as long as they are stored in a clean environment and patients are prepped after their use, is a safe practice that does not affect surgical site sterility, as this had been previously reported by Rooney et al. in 2008 [5].

Our literature search yielded 16 articles. For our inclusion criteria, we searched for studies that were published in the year 2000 and afterwards. We searched for publications that discussed surgical marking pens, contained English text, were experimental studies, review papers, retrospective studies, and prospective studies. We excluded publications that were commentaries, editorials, or articles that did not have full text available. Studies were evaluated by two reviewers. After removing duplicate articles, we identified 16 articles, of which three articles were removed as they were not relevant to surgical marking pens and colonization or infection. After full-text review, one additional study was excluded as it discussed a marking method other than a marking pen. Three additional papers were included after reference reviews. In total, 16 papers were included in our analysis. There were five references that are referenced in the review but were not included in our analysis.

## Materials and methods

**Figure 1:**
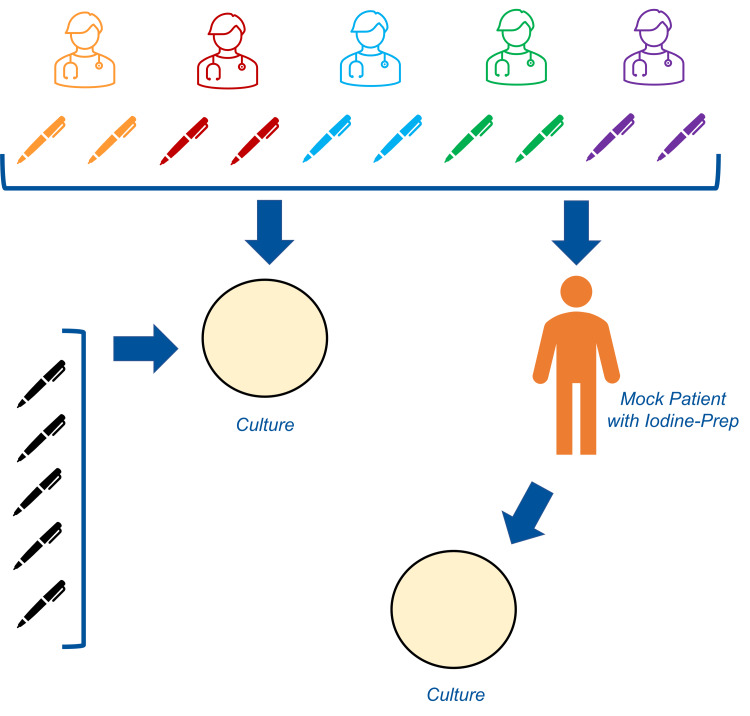
Pictographic demonstration of laboratory study design.

Two multiply used marking pens from five different attending plastic surgeons at a single institution were cultured in standard fashion for aerobic and anaerobic growth. All pens were used repeatedly in office settings for performing patient markings. The different attending surgeons operate within the same health system but have different office locations. All of the marking pens were wide-tipped permanent ink delivery instruments. Those same 10 marking pens were then used to mark incision sites on mock patients. Standard povidone-iodine prepping was then performed in a paint-only fashion over the skin markings, and cultures were again taken. Lastly, a control group was formulated consisting of cultures from five sterile pens from the operating room. Each sterile pen was opened, uncapped, and then swabbed. All twenty-five cultures were analyzed in the hospital laboratory in a blinded fashion, and the lab personnel were not aware of whose pens were sampled. All cultures were performed with nonselective media and tested in standard procedure for bacterial and fungal growth (Figure 1).

We performed a literature search in PubMed using the Boolean search phrase: surg* marking pen OR "surgical site marking" AND "infection.” We included articles that were published on the colonization of marking pens or studies pertaining to marking practices and SSIs. Additional articles were identified by searching the references of the articles from our preliminary search to expand our list of included publications. Preliminary abstract screenings were performed with secondary full-text article reviews to evaluate article relevance.

## Results

The analysis of culture data was performed, with 10 direct pen swab cultures being one group, 10 marked patients post-prepping serving as a second group, and five control pens constituting the third. The five control pens, consisting of cultured sterile markers, revealed no bacterial growth. The 10 direct pen cultures revealed no bacterial growth on seven accounts. Two samples grew colonies of coagulase-negative staphylococci and one culture contained *Pseudomonas aeruginosa*. The 10 experimental samples from marking mock surgical patients resulted in eight negative cultures and two positive cultures with coagulase-negative staphylococci. Although *Pseudomonas* was detected on standard pen culture, no pseudomonal growth was present in any of the samples after patient marking and prepping with povidone-iodine.

## Discussion

Historically, personal marking pens were utilized by surgeons with the explanation that pens are kept clean with their personal belongings and surgical prep after patient marking would prevent the spread of infection via this “pen vector.” Some of the cultured pens in our study grew *Staphylococcus* and *Pseudomonas*, indicating that various organisms can and do survive on the marking pens. In a similar study by Ridley et al. [6], it was found that 15% of surgical marking pens used in anterior cruciate ligament (ACL) reconstruction surgeries were contaminated with *Staphylococcus*. In another study of ACL reconstruction surgeries, Nakayama et al. demonstrated that 6% of patients had positive skin colonization near the incision site and 2% had positive graft colonization with *Staphylococcus epidermidis*, indicating that a surgical pen could have transmitted *S. epidermidis* from colonized skin to the graft [7]. In an experiment culturing pens used in shoulder reconstruction surgery, one of the 43 studied pens demonstrated growth of *Propionibacterium acnes* [8]. A literature review of surgical site markers as sources of infection determined that markers can be a source for cross infection with MRSA, other bacteria, fungi, or viruses [9]. Thus, surgical marking pens could conceivably be the vector of bacterial transmission from one patient to another.

An important factor to consider is the type of surgical marker used, as surgical subspecialties differ in their preferences for skin marking instruments. Breast lesion stickers are commonly used to localize breast lesions in surgical oncology [10]. Radiation oncologists prefer the use of permanent tattoos with India ink [11]. Due to the nature of plastic and reconstructive surgery, clarity of skin marking is essential for the surgical planning of meticulous procedures and optimum cosmetic results [12,13]. This study examined bacterial growth on wide tipped permanent ink delivery instruments, which is the standardized practice for skin marking of plastic and reconstructive surgery procedures at our institution. An experiment by Bathla et al. evaluated the ability of commonly used skin markers to withstand surgical skin preparations and found that Sharpie black permanent marker was the best performer across all Fitzpatrick skin types [14]. Permanent marker contains ethanol-based ink that is bactericidal against MRSA. “Fresh” pen tips are recommended, as old, desiccated marker pens can harbor pathogens [15]. Ballal et al. compared the risk of cross-infection with the use of dry white board marker versus permanent marker at various time points. It was found that 100% of dry white board markers had positive cultures after 10 minutes, whereas the risk of infection decreased significantly with time in permanent markers. Thus, it is recommended that permanent marker be used between patients at intervals greater than 10 minutes to decrease infection risk [16]. A similar study by Sim et al. found that the survival time of MRSA on marker pens decreased with time, thus supporting a prolonged time interval of pen usage between patients [17].

It is also important to consider the type of skin preparation used to sterilize the surgical area. Iodine-based prep preserves surgical skin markings better than chlorhexidine-based prep. This may be attributed to differences in solution application in accordance with manufacturer guidelines, as povidone-iodine prep involves painting a single layer without scrubbing and chlorhexidine prep involves repeated forward and backward strokes for 30 seconds [18]. In 2008, Rooney et al. performed an experiment where they cultured forearms that had been marked like a simulated surgical site with a multiply used marking pen and found that there was no bacterial growth following site sterilization with a 10% povidone-iodine solution [5]. Another experiment used cultured marking pens on surgical sites that were prepped with betadine (povidone-iodine) scrub followed by betadine (povidone-iodine) paint. After 72 hours, all cultures were negative, indicating that the sterility of the surgical field was not compromised [19]. The active ingredient in betadine, povidone-iodine, has a published spectrum of killing nearly all bacteria in 15-30 seconds, including *Staphylococcus* and *Pseudomonas*. No bacterial resistance has been reported to date [20].

Our study is limited by its laboratory design and small sample size. There is a further need for repeated studies with larger sample sizes. Our results are insufficient to establish an association between utilizing a multiply used pen and SSIs, nor is it sufficient to establish that single-use pens decrease the incidence of SSIs. The ideal study design to establish a transference of bacteria from a multiply used marking pen would be to swab the 10 patients after prepping with betadine and to re-swab them after drawing with a marking pen. Future efforts should also focus on elucidating the relationship, if any, between SSI and marking pen reuse.

Despite its limitations, the results of this study hold relevance to antisepsis. While colonization is not sufficient to determine if multiuse or nonsterile marking pens increase the rate of SSIs, our findings support that additional research is warranted with larger sample sizes. While the Joint Commission aims to reduce HAIs as Goal 7 of their National Patient Safety Goals, the recommendation described in the Universal Protocol for Preventing Wrong Site, Wrong Procedure, and Wrong Person Surgery™ does not provide specific recommendations as to the type of tool used for preoperative marking. They do not make a specific recommendation to use sterile marking pens or to marking pens only once [21]. However, the Joint Commission’s required markings are not usually incised with the scalpel, as are the markings in plastic surgery. Rather, these markings are usually at some distance from the incision site, denoting the correct site or laterality of the procedure to be performed. On the contrary, aesthetic surgery patient markings indicate areas for incisions; thus, the surgeon’s scalpel is cutting directly through the ink marking. The popularity of outpatient plastic surgery continues to increase, and concerns over the incidence of infections in an outpatient setting have been raised. However, the rate appears to be relatively low in experienced hands, with major SSI incidence of 0.46% following aesthetic surgery [22] and 0.0781% specifically in ambulatory plastic surgery centers [23]. Regardless, SSIs are a significant preventable cause of morbidity and mortality, and every effort should be taken to reduce the risk of infection.

## Conclusions

Based on this experimental study and literature review, we assert that it is safest and, hence, the best practice to mark patients with a sterile pen and use it only once. In doing so, one has effectively removed the possibility of transferring organisms from one patient to another and one does not have to rely on the surgical prep to destroy cross-inoculated organisms of an iatrogenic variety. We identified that multiply-used surgical marking pens showed evidence of coagulase-negative staphylococcal and pseudomonal colonization. Our findings affirm that marking pens may serve as a nidus for bacteria, and we recommend that single-use surgical marking pens be standard practice at every institution with adequate resources.
